# A Microphysiological System with an Anaerobic Air-Liquid Interface and Functional Mucus Layer for Coculture of Intestinal Bacteria and Primary Human Colonic Epithelium

**DOI:** 10.1002/admi.202400093

**Published:** 2024-06-19

**Authors:** Raehyun Kim, Nancy L. Allbritton

**Affiliations:** Department of Biological and Chemical Engineering, Hongik University, Sejong-si 30016, Republic of Korea; Department of Bioengineering, University of Washington, Seattle, WA 98195, USA

**Keywords:** air-liquid interface, intestinal anaerobic bacteria, intestinal model system, microphysiological system, mucus, oxygen gradient

## Abstract

Coculture of intestinal bacteria with primary human intestinal epithelium provides a valuable tool for investigating host-colon bacterial interactions and for testing and screening therapeutics. However, most current intestinal model systems lack key physiological features of the in vivo colon, such as both a proper oxygen microenvironment and a mucus layer. In this work, a new in vitro colonic microphysiological system is demonstrated with a cell-derived, functional mucus that closely resembles the in vivo colonic mucosa and apical microenvironment by employing an anaerobic air-liquid interface culture. The human primary colon epithelial cells in this new in vitro system exhibit high cell viability (>98%) with ≈100 μm thick functional mucus layer on average. Successful coculture of model anaerobic gut bacterial strains *Lactobacillus rhamnosus GG* and *Anaerobutyricum hallii* without loss in human cell viability demonstrates that this new model can be an invaluable tool for future studies of the impact of commensal and pathogenic bacteria on the colon.

## 1. Introduction

The human intestine forms one of the most extensive interfaces interacting actively with the outer environment. The intestine digests dietary materials and absorbs nutrients, ions, and water. The majority of commensal microbes in the human body are found in the intestine, where they interact with the host to regulate intestinal homeostasis and influence the physiology of the entire host. Intestinal microbes educate the host immune system,^[1]^ modify host metabolism,^[2]^ modulate the pathogenesis and progression of diseases, and alter therapeutic responses.^[3]^ In the healthy colon, an extremely low level of apical O_2_ (<1%) establishes an environment suitable for the growth of obligate anaerobes.^[4]^ A mucus layer prevents direct contact between the colonic epithelium and intestinal microbes while providing binding sites and acting as a nutrient source for many colonic bacteria.^[5]^ The high viscosity (≈0.4 Pa·s)^[6]^ and small pore size (0.5 μm)^[7]^ of the mucus further act to protect the intestinal epithelium by slowing the diffusion of bacterial toxins and inflammatory mediators.^[8]^ The mucus layer with its associated microbes form a complex ecosystem. A comprehensive understanding of this dynamic network of host-gut microbial interactions is required for a better understanding of the healthy and diseased states of the human colon.

In vitro intestinal model systems have unique advantages over animal models for studying host-gut bacterial interactions. They enable control over experimental variables and environment with fewer ethical concerns and lower costs. Moreover, considering the significant interspecies differences, in vitro human models are expected to provide experimental findings more relevant to humans than that of model animals. In developing an in vitro colonic model system for anaerobe gut bacterial coculture, critical features to recapitulate are the O_2_ microenvironment, mucus layer, in addition to high-resistance epithelial barrier, basal-apical cell polarization, and cell composition. A readily accessible apical and basal compartment or reservoir further enhances the model system enabling sampling from or addition to the two epithelial sides. Several model systems with a subset of these features including either the anaerobic lumen or mucus layer have been reported.^[9]^ A few studies possessed both an anaerobic apical compartment, a cell-derived mucus layer, and primary human cells using commercially available hanging basket culture inserts.^[9d,e]^ These systems used an O_2_ impermeable stopper to block O_2_ ingress^[9d]^ or by flowing de-oxygenated fluid flow^[9e]^ to ensure that the apical side of the tissue remained at a low O_2_ saturation. Both strategies demonstrated bacterial coculture with at least a single strain of intestinal bacteria, but the mucus layer was much thinner than that of the in vivo colonic mucus. Moreover, these models are still physiologically distant from the in vivo healthy colon, where the epithelium interfaces with a mucus layer and apical gas or solids, in that the epithelium was immersed under a liquid.

Air-liquid interface (ALI) culture has been used since the 1950s for culturing the epithelial cells of organs that face gas phases in vivo, such as skin,^[10]^ respiratory organs,^[11]^ or reproductive organs.^[12]^ In these systems, ALI cultures induced a more in vivo-like differentiated phenotype, including higher cell density, increased cell height, mucus production, and cilia formation.^[13]^ ALI culture has also been introduced to intestinal cells cultured on a stiff porous membrane and yielded a mature epithelium with differentiated columnar cells.^[14]^ In one of these studies, a return to submerged culture converted the cell morphology to a squamous phenotype.^[14f]^ Intestinal cultures under ALI also possess significantly increased goblet cell numbers and mucus layer thickness compared to submerged cultures.^[9b]^ When supplemented with vasoactive intestinal peptide (VIP) on the basal tissue side, ALI culture of the intestine generates a bacteria-impenetrable apical mucus with a thickness of greater than several hundred microns.^[9b]^ In this instance, VIP greatly increased the density of goblet cells in the epithelium as well as the amount of mucus secreted. An added benefit was the ability of VIP to modulate salt/water balance across the epithelial cells so that the mucus layer was fully hydrated. While these systems represent impressive accomplishments, a high apical O_2_ tension is not a physiologic feature of the in vivo colon in which the apical-bordering epithelial cells live under a physiologic hypoxia (<1% O_2_). Additionally, these systems are inappropriate for the coculture of the colon’s commensal obligate anaerobes since O_2_ is toxic to most of these microbes.

Here we demonstrate a new in vitro colon model that possesses an anaerobic apical surface and an oxygenated basal surface replicating the in vivo physiologic O_2_ environment. Human primary colon epithelial cells were initially grown in a submerged culture medium in a hanging basket format, then moved to an aerobic ALI culture, and finally to an anaerobic ALI culture. Apical O_2_ was depleted by cellular O_2_ consumption and blocking O_2_ ingress into the apical reservoir. Transport properties through an optimized porous membrane are measured as well as consumption of nutrients and secreted cytokines by the epithelial cells. The mucus layer formed was evaluated for its thickness and functionality. The performance of the cultures was characterized during coculture with two commensal colonic bacteria, a facultative anaerobe *Lactobacillus rhamnosus GG* and an obligate anaerobe *Anaerobutyricum hallii*.

## 2. Results

### 2.1. Optimization of the Polyester (PET) Porous Membrane for Cell Culture Under ALI

Initially, we sought to combine two previously developed systems, one of which generated an anaerobic apical compartment and the second which supported the formation of a thick apical mucus layer.^[9b,15]^ The goal was to create a mucus-covered colonic epithelium with a de-oxygenated apical compartment for the culture of obligate anaerobes. Both innovations utilized a hanging basket format and so physically appeared facile to integrate. The O_2_-gradient cassette forms an O_2_ gradient across a colonic epithelial monolayer by enclosing the apical reservoir with tightly sealed O_2_ impermeable walls while O_2_ remains freely accessible to the basal reservoir.^[15,16]^ Cellular O_2_ consumption depletes O_2_ from the apical compartment while the living epithelium consumes O_2_ diffusing to the basal surface via the basal reservoir.^[15,16]^ Primary colonic epithelial cells were cultured in the gradient O_2_ cassette to form a differentiated epithelium comprised primarily of colono-cytes as described previously. The apical fluid was then removed (to initiate ALI) and this compartment was then sealed to prevent ingress of O_2_. Over 2 days, most of the epithelial cells visibly sloughed from their culture surface, leaving large open gaps in the epithelial monolayer (Figure S1A, Supporting Information). Despite the loss of a functional epithelial cell monolayer, the O_2_ in the apical compartment decreased over time likely due to O_2_ consumption by the remaining monolayer cells or living but detached cell clumps in the apical reservoir (Figure S1B, Supporting Information).

In the case of ALI, all nutrients must access the cells through the basal reservoir since there is no apical medium. The transparent PET membrane with 0.4 μm (PET0.4) used in this study is the most frequently used porous membrane for epithelial cell cultures. But notably, this porous membrane has less than 1% open area for transport (Table 1), potentially reducing the nutrient access to the apical side. Aiming to increase the nutrient transport window, we tested another porous membrane with a larger open area. PET membrane with 8 μm pores (PET8) possesses 16.7 times larger openings than PET0.4 (Table 1), reasonably inferring improved nutrient transport from the basal tissue side. However, despite the increased open area, the cells were not able to form a confluent monolayer on PET8 even when fully submerged (Figure S1C, Supporting Information). Some cells were able to attach to the substrate but failed to proliferate and became squamous in morphology instead of columnar, the in vivo shape of colonic epithelial cell.^[17]^ It is possible that the lower pore density (20 times less than the 0.4 μm PET membrane), despite a larger transport window per pore, may expose only the cells near the pores to the basal medium with nutrients and growth factors.

**Figure 1.**
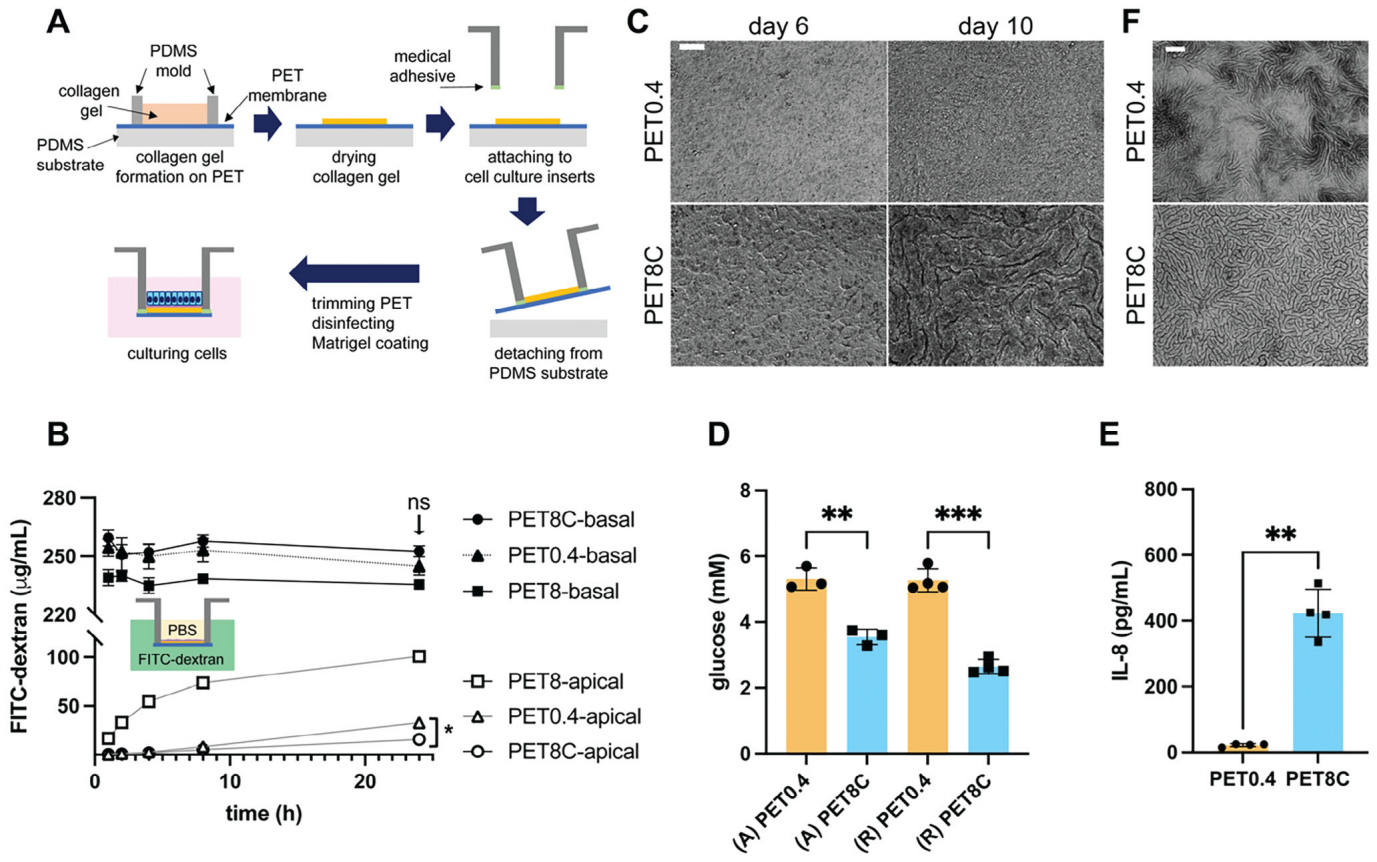
Development and characterization of the PET8C culture surface. A) Preparation of PET8C. B) Diffusion of FITC-dextran (40 kDa) through the porous membrane without cells. Statistical analysis results are indicated for 24 h data. n = 4 for each. Two-way ANOVA with Geisser-Greenhouse correction was performed. * indicates *p* < 0.05, “ns” not significant. C) Brightfield images of the cells grown on PET0.4 and PET8C in submerged culture. Scale bar = 100 μm). D) Glucose levels in the basal medium after 1 day (A) or 2 days (R) of ALI culture. (A) and (R) indicate Advanced DMEM/F12 and RPMI used as a base medium, respectively. n = 3 for (A) and 4 for (R). Brown-Forsythe and Welch One way ANOVA test was performed for the statistical analysis. **, *** indicates that *p* < 0.01 and *p* < 0.005, respectively. E) IL-8 levels of the basal media of the cells after two days in ALI culture. n = 4. Brown-Forsythe and Welch One way ANOVA test was performed for the statistical analysis. ** indicates *p* < 0.01. F) Brightfield images of the cells on PET0.4 or PET8C after 3 days of ALI culture. Scale bar = 200 μm.

To obtain high cell viability while exploiting the large open area of PET8, we introduced a thin collagen layer onto PET8 and named as PET8C (Figure 1A). This thin collagen layer (≈5 μm thick) functions as a physical support for the cells throughout the entire culture area. The open structure and largely aqueous composition of the collagen hydrogel is also expected to support lateral movement of nutrients within the collagen layer, enhancing cell access to nutrients. Colonic epithelial cells cultured on the PET8C formed a contiguous monolayer across the surface of the collagen visually similar to that formed on PET0.4 (Figure 1C day 6). Next, the ability of the epithelial cells to form a monolayer on PET8C under aerobic ALI conditions was assessed. A contiguous monolayer was formed under aerobic ALI conditions for cells on PET8C that was visually similar in appearance to that grown on PET0.4 under these conditions as well (Figure 1C day 10).

We validated and compared biochemical transport through PET8C with that through PET0.4 in the absence of cells. Basal-to-apical transport of a surrogate molecule, 40 kDa FITC-dextran (40 kDa), similar in size to key growth factors for colonic epithelial cells, was measured for PET0.4, PET8, and PET8C. Notably, FITC-dextran concentration in the apical compartment was significantly higher in PET8 than in PET0.4 throughout the measurement, confirming a larger open area of PET8 compared to PET0.4 (Figure 1B). At 1 h, the apical concentration of the FITC-dextran was 50 times higher with PET8 than with PET0.4 (16.81 ± 6.12 μg mL^−1^ vs 0.34 ± 0.19 μg mL^−1^). The difference diminished over time, but the apical concentration of the FITC-dextran was still three times higher in PET8 than in PET0.4 (100.80 ± 4.81 μg mL^−1^ vs 32.78 ± 3.09 μg mL^−1^) at 24 h of diffusion. As expected, FITC-dextran diffuses through PET8 much more efficiently than through PET0.4. The apparent permeability ($P_{\text{app}}$) of PET0.4 and PET8 for 40 kDa FITC-dextran calculated from the measurement were 9.4×10^−9^ m ^−1^s and 8.4×10^−8^ m ^−1^s, respectively. Meanwhile, the apical concentration of FITC-dextran was not significantly different between PET0.4 and PET8C at times up to 8 h (Figure 1B). By 24 h, the apical concentration with PET8C was about half of PET0.4 (32.78 ± 3.09 μg mL^−1^ vs 15.77 ± 4.91 μg mL^−1^), while basal concentrations were statistically not different (PET0.4: 245.07 ± 4.83 μg mL^−1^ vs PET8C: 252.65 ± 2.85 μg mL^−1^). The $P_{\text{app}}$ estimated from the measurement for PET8C was 4.3×10^−9^ m ^−1^s, which is 45% of the $P_{\text{app}}$ for PET0.4 and comparable to the estimated $P_{\text{app}}$ values for apical-to-basal diffusion of 40 kDa FITC-dextran based on the linear relationship between $P_{\text{app}}$ and reciprocal of Stokes radius from previous reports of movement through a collagen gel on PET porous membranes.^[18]^ Thus the collagen layer likely hindered the bulk or averaged movement of FITC-dextran through the PET8C surface. The improved cell growth on the PET8C compared to PET8 may be due to multiple factors and not simply differences in nutrient movement. For example, the thin collagen layer presents a less stiff surface (≈55 kPa)^[19]^ compared to either ECM-coated porous membranes (1.5 MPa),^[20]^ which may have acted in concert with pore distribution/sizes to enhance cell health and growth.

To further investigate cell growth on the PET8C, we compared the concentrations of biochemicals consumed or produced from the cells grown on PET0.4 and PET8C in the aerobic ALI condition to evaluate whether the collagen layer hampers the transport of biomolecules to and from the cells. First, the basal glucose level was measured after 1 or 2 d under ALI on PET0.4 and PET8C. The culture medium with Advanced DMEM/F12 as a base medium contained 11.42 mM glucose. After 1 d, the measured glucose level in the basal medium of the cells cultured on PET8C was 3.55 ± 0.23 mM and 5.31 ± 0.20 mM for those on PET0.4 (Figure 1D). When the cells were cultured in RPMI medium (6.33 mM glucose which is commonly used for coculturing with immune cells) 2 d, the glucose level in the basal medium was measured at 2.64 mM for cells on PET8C and 5.27 mM for cells on PET0.4. These data indicate that the collagen layer did not limit the glucose transport to the cells, which is also evidenced by the healthy cell morphology shown in Figure 1C. We also measured the levels of a cell-produced cytokine, IL-8, in the basal media of the cells grown on PET8C and PET0.4 secreted for one day under ALI culture, since IL-8 is constitutively secreted by colonic epithelial cells.^[21]^ IL-8 levels were 7.7 times higher in the basal medium of the cells grown on PET8C than on PET0.4 (Figure 1E), again indicating that the collagen layer did not impair the basal transport of secreted proteins. Additionally, the IL-8 level for the cells on the PET8C under ALI was 2–3 times higher than that measured previously in the basal reservoir of fully submerged, healthy colonic monolayers but much lower (10 times) than that in inflammatory condition.^[21]^ These data suggest that the PET8C successfully supports colonic epithelial cell viability under aerobic ALI culture.

### 2.2. Formation of an Anaerobic ALI Culture Model

**Figure 2.**
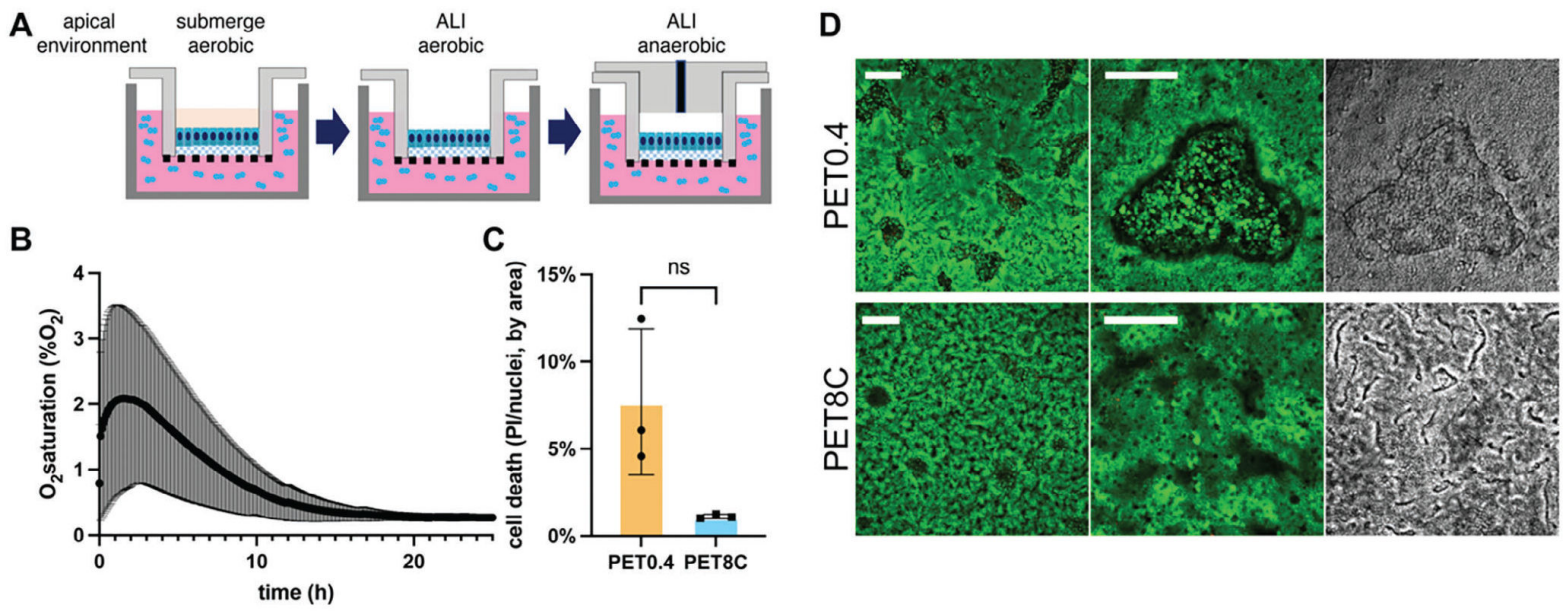
Creation of an anaerobic ALI culture. A) A schematic diagram for the procedure of creating an anaerobic ALI environment. B) O_2_ profile of the apical gas in the anaerobic ALI measured from 5 independent cultures. C) Cell death estimated by measuring the PI+ area and dividing by the area positive for Hoechst 33342 (Y axis). n = 3. An unpaired t-test with Welch’s correction (for non-equal standard deviations of each sample) was used for the statistical analysis. ns indicates statistically not significant. D) Fluorescence and DIC microscopy images of anaerobic ALI cultures. The left four panels are fluorescence microscopy images in which green indicates Calcein-AM staining live cells and red PI labeling dead cells. The right two panels are DIC microscopy images. Scale bar = 500 μm for the left column, 200 μm for the center and right columns.

Next, we tested whether PET8C supports an anaerobic ALI culture of primary colon epithelial cells. Cells were grown to confluence as a submerged culture for 6 days on a PET8C membrane, and then the apical medium was removed to create an aerobic ALI culture. After 4 days, the cell culture was converted to an anaerobic ALI system. The cells on the culture inserts were placed in an anaerobic chamber to eliminate O_2_ from the apical reservoir, and an oxygen impermeable plug (O_2_ gradient cassette) was installed in the apical reservoir to block apical gas exchange. (Figure 2A). Then the cell culture inserts with the plug installed were brought out of the anaerobic chamber and placed into a CO_2_ incubator. Under these conditions, the basal reservoir is open to the surrounding atmosphere and remains oxygenated. The apical reservoir remains anaerobic due to the initial removal of O_2_, the blockage of O_2_ ingress by the O_2_ impermeable apical reservoir walls, and the ongoing O_2_ consumption of the epithelial cells.^[15]^ The O_2_ level of the apical reservoir was initially below 2% and further decreased below 1% within 18 h (Figure 2B). Under these anaerobic ALI conditions, cell viability on the PET0.4 and PET8C surfaces was not statistically different (Figure 2C), but patches of damaged epithelium were observed on PET0.4 (Figure 2D). This partial cell monolayer does not represent the healthy epithelium and would permit the ingress of gut bacteria through the epithelium infecting the underlying tissue and stimulating an immune response.

### 2.3. Formation of a Mucus Layer on Epithelial Cells Under an Anaerobic ALI Culture

**Figure 3.**
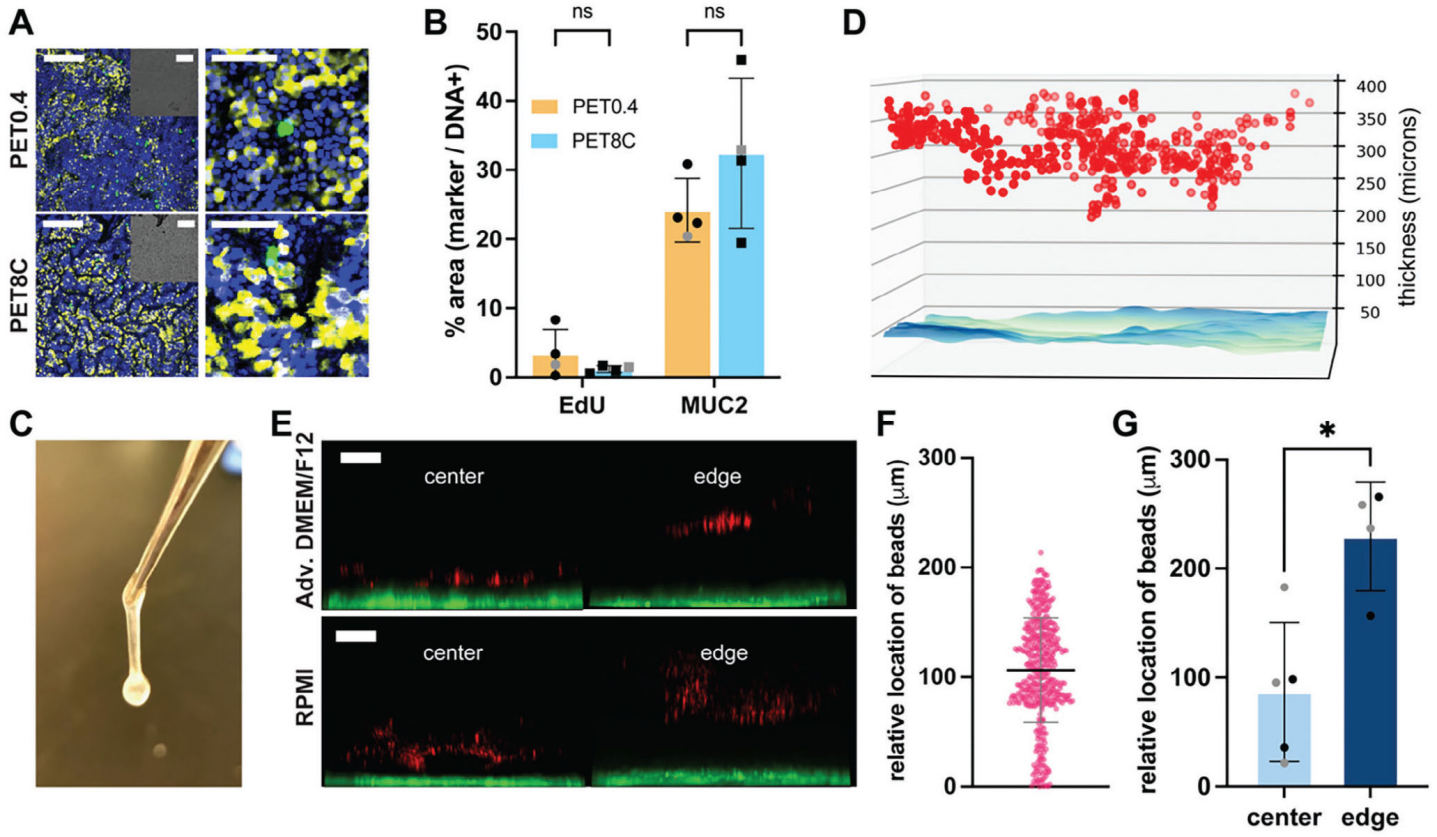
Characterization of mucus formation by monolayers under anaerobic ALI culture. A) Representative fluorescent images of the cells cultured for 2 days under the anaerobic ALI. Green: EdU, yellow: MUC2, blue: Hoechst 33 342. Scale bar = 200 μm for the left images, including the inset, and 50 μm for the right images of each condition. B) EdU-incorporating proliferative and MUC2-positive goblet cell populations in the cells cultured for 2 days under anaerobic ALI. Unpaired Welch’s t-test was used for the statistical analysis, with ns indicating “not significant”. Data in black (n = 3) were obtained from donor 1 and grey (n = 1) from donor 2 cells. C) The mucus collected using forceps after 2 days of anaerobic ALI culture. D) A representative reconstruction image of the cell surface (blueish plane) and the locations of the beads (red dots). E) XZ projection images of the fluorescent beads overlaid on the cells. Red indicates 1 μm fluorescent beads and green Calcein labeled live cells. Scale bar = 100 μm. F) An example of relative locations of the fluorescent beads collected from a culture. Each spot corresponds to a bead at the location detected relative to the cell surface. At least 240 beads from 2–4 areas were imaged. More data from independent samples are found in Figure S2 (Supporting Information). G) The average relative locations of the fluorescent beads at the center and near the edge. Black (n = 2) and grey (n = 3 for center and 2 for edge) data indicate the samples with Advanced DMEM/F12 and RPMI, respectively, as a base medium. * indicates *p* < 0.05 when a nonparametric, unpaired Mann-Whitney test was used.

The presence of proliferative and goblet cells in cultures on the PET0.4 and PET8C surfaces was evaluated. Monolayers on both the PET8C and PET0.4 possessed EdU-positive, i.e., proliferative cells (Figure 3A), with no statistical difference in relative populations between the cultures on the two membranes. No statistical difference in the amount of mucin 2 detected by immunofluorescence was identified, suggesting that the cultures possess similar numbers of goblet cells. Notably, staining for mucin-2 was much greater (>20%) than that previously reported for submerged anaerobic cultures,^[15,16,22]^ and similar to that reported previously for aerobic ALI cultures.^[9]^ Monolayers cultured on PET8C under anaerobic ALI also display a thick, viscous coating that is picked up by forceps (Figure 3C). To determine the mucus layer thickness, 1 μm-sized fluorescent beads were overlaid onto the cell cultures (Figure S2A, Supporting Information). Most microbeads could not penetrate the mucous layer and remained at a distance above the epithelial cells (Figure 3C,D). The thickness of the mucus layer depended on the location in the culture, with a thinner mucus layer at the culture center and the thickest layer near the edge (Figure 3D,E; Figure S2B, Supporting Information). This mucus layer was significantly thinner than that of previously reported mucus produced by cultures under aerobic ALI.^[9]^ When growth factors were removed from the basal reservoir of anaerobic ALI PET8C cultures, a thicker mucus was generated (300 μm, Figure S2D, Supporting Information) but at the cost of reduced cell viability. We found no statistical difference in the mucus layer thickness between the cells cultured in RPMI and Advanced DMEM/F12 (Figure 3G, grey and black, respectively), but local epithelial damage was detected when RPMI was used as a base medium (Figure S2E, Supporting Information). In summary, these data indicate that the PET8C supports the formation of a mucus layer under anaerobic ALI culture conditions, unlike the PET0.4 surface.

### 2.4. Gut Bacterial Coculture in the Anaerobic ALI

**Figure 4.**
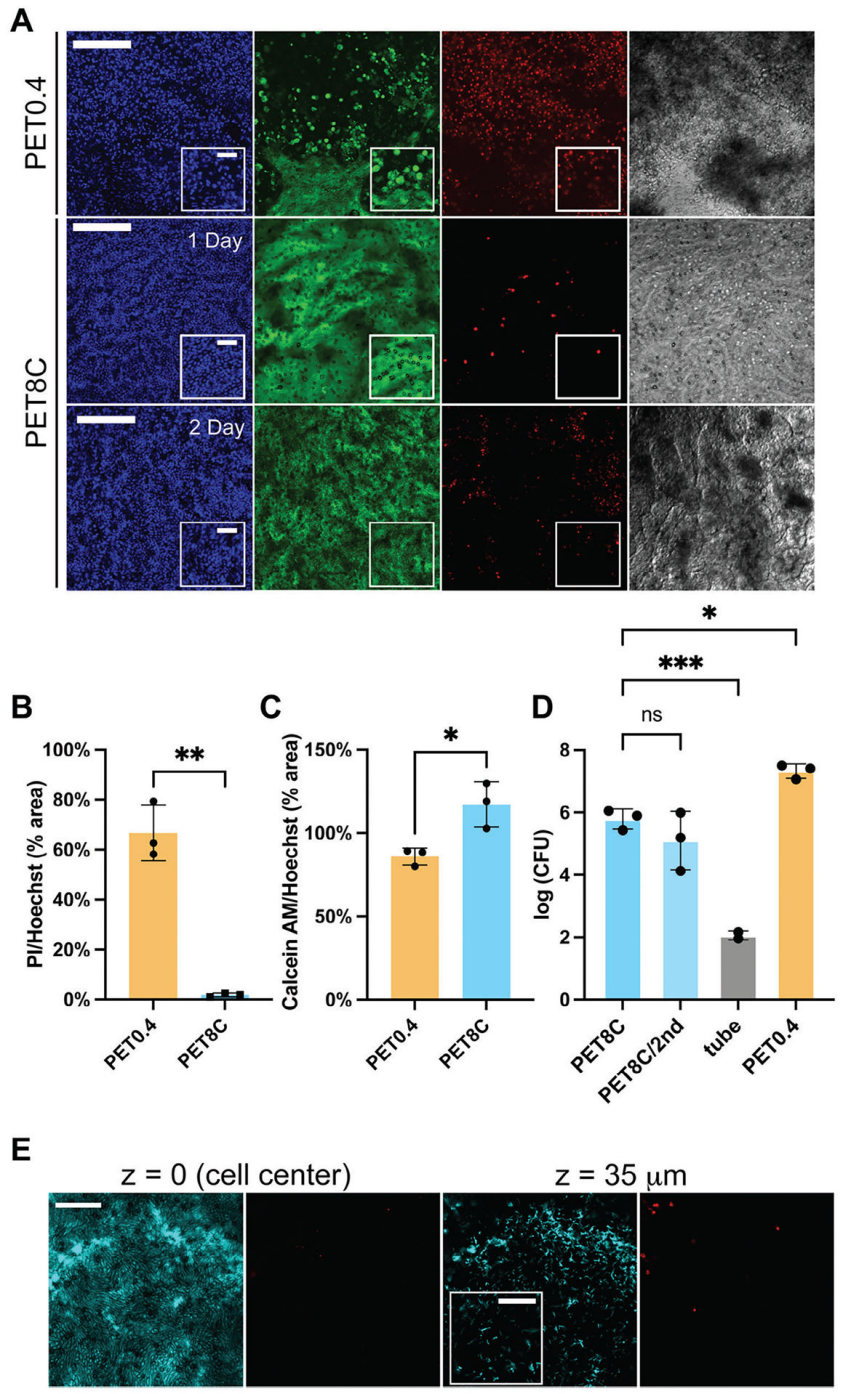
Coculture of human colonic epithelium with anaerobic bacteria. A) Representative images of the human cells cultured on PET0.4 or PET8C after 1 or 2 d of coculture with LGG under anaerobic ALI. Hoechst 33342 (blue) labels DNA of both living and dead cells. Calcein (green) is retained in the cytoplasm of live cells after metabolism of Calcein AM. Propidium iodide (red) labels the DNA of dead cells. Scale bar = 200 μm for the larger images and 50 μm for the insets. Top: cells on PET0.4 cocultured with LGG for 1 d, middle: the cells on PET8C cocultured with LGG for 1 d, bottom: cells on PET8C at 2 d cocultured with LGG and inoculated twice with a 1 d interval. B) Cell death was measured by dividing the PI-positive area by the Hoechst 33 342 positive area. n = 3. C) Live cells were assessed by measuring the Calcein positive area divided by the Hoechst 33 342 positive area. n = 3. D) Growth of bacteria in coculture and corresponding monoculture without human cell exposure (tube). Three technical replicates were used for cocultures and two for the monoculture (tube). For statistical analyses, unpaired Welch’s t-tests were performed for C and D, and an ordinary one-way ANOVA was performed for E after confirming that the standard deviations are not significantly different using an F-test. *, **, *** indicate *p* < 0.05, <0.01, and <0.005 respectively. E) Representative images of the *LGG* cocultured with human colonic epithelial cells on PET8C. Nucleic acids of bacteria and human cells were stained with Syto9 (cyan, left panels) and dead cells with PI (red, right panels) at 2 different Z planes above the nuclei of the epithelial cells. Scale bar = 100 μm for the larger images and 20 μm for the inset.

Finally, we evaluated whether this new model could be used for bacterial coculture. A facultative anaerobic probiotic strain, *Lactobacillus rhamnosus GG (LGG)* is frequently used for bacterial coculture studies since it is a common resident of the colon. *LGG* was cocultured in the anaerobic ALI condition with the human primary colonic epithelial cells grown on PET0.4 and PET8C. After 24 h of *LGG* coculture, large numbers of dead cells were present in the cultures on the PET0.4 with few remaining attached cells (Figure 4A, Figure S3A, Supporting Information). On the contrary, for the cells on PET8C, the epithelial integrity was maintained with high cell viability (Figure 4A, Figure S3A, Supporting Information). The colon epithelial cells on PET8C exhibited significantly higher viability after 24 h with *LGG* coculture than those on PET0.4 (Figure 4B,C). The number of viable *LGG* estimated by counting the colony-forming units (CFU) increased 1000-fold during the 24 h coculture (from ≈10^3^ CFU at t = 0 to ≈10^6^ CFU at 24 h), indicating that this platform supports robust *LGG* growth with the human primary epithelial cells (Figure 4D). For comparison, the growth of *LGG* 24 h under conventional bacterial culture without human cell coculture was significantly slower than the anaerobic ALI coculture (Figure 4D). Live/dead staining also demonstrated that the *LGG* were viable 24 h coculture (Figure 4E). However, the distance between the *LGG* and the human colon epithelium after 24 h was smaller than the mean mucus thickness measured with the fluorescent beads, suggesting that the mucus layer is damaged during bacteria inoculation or partially penetrable to bacteria. The high viability of the cells cocultured on the PET8C allowed an examination of whether PET8C supports consecutive inoculation, mimicking the emptying and regrowth of the gut bacteria in vivo. The *LGG* cocultured was washed out at 24 h, and then the same amount of *LGG* (10^3^ CFU) was inoculated for the second round. Despite the increase of dead cells compared to those at 24 h coculture, an intact epithelial layer was maintained (Figure 4A, bottom row). *LGG* grew after the second inoculation to a similar extent in the coculture as it did during the first round of coculture (Figure 4D). Last, we tested whether this platform supports the coculture of *Anaerobutyricum hallii (A. hallii)*, an obligate anaerobic probiotic strain found in the human colon. Almost all *A. hallii*, as well as the human cells, were viable after 24 h (Figure S3B, Supporting Information). These data demonstrate that this new anaerobic ALI model can be used for stable gut bacterial coculture.

## 3. Discussion

We demonstrate a new intestinal model that possesses a mucus layer in the anaerobic lumen. First, we exploited an ALI to induce robust mucus layer production based on our previous work showing that more goblet cells are expressed in an ALI culture than in a submerged culture leading to the formation of a cell-derived mucus layer.^[9b]^ In typical ALI culture, direct contact with the atmospheric air on the apical side increases O_2_ flux compared to conventional submerged culture, resulting in alterations of the intestinal epithelial cell behavior with a bias toward differentiation and maturation^[14b]^ and also decreased HIF-1α expression, reduced glycolysis, and increased oxidative phosphorylation with higher cytochrome c oxidase (COX) activity.^[14d]^ An ALI culture of the murine primary intestinal epithelial cells generated the self-organized epithelium with properly differentiated phenotypes surrounding Ki67 expressing proliferative cells, which is reminiscent of the cell organization within a crypt in vivo.^[14f]^ Previously, we also showed that the cell-derived mucus could be generated in ALI culture of human primary colonic epithelial cells in the presence of VIP.^[9b]^ Collectively the previous research suggests that an ALI culture promotes the in vivo-like homeostasis of the intestinal epithelial cells by enhancing O_2_ delivery to the cells.

However, an ALI culture does not reflect the O_2_-deprived apical environment of the human small and large intestines in vivo. To generate an anaerobic apical environment, we depleted the O_2_ in the apical side of an ALI culture using as a starting point the simple system that we previously developed to generate an O_2_ gradient in submerged culture.^[15]^ Our data indicate that PET0.4, the most frequently used porous membrane for epithelial culture, failed to sustain cell viability under an anaerobic ALI condition. Increasing the total open area to 5% by substituting PET0.4 with PET8 did not improve the cell viability under the anaerobic ALI, most likely due to the inhomogeneous basal transport from 20 times lower pore density (1×10^5^ cm^−2^).

To improve cell viability in an anaerobic ALI culture condition, we introduced an additional hydrogel layer on PET8. This additional collagen layer on the PET8 membrane allows lateral distribution of biochemicals while exploiting the larger open area of PET8. The collagen layer significantly slows down the transport of FITC-dextran, but transport through PET8C was comparable to PET0.4 for at least up to 8 h, then less FITC-dextran permeated through PET8C than PET0.4. We show first in the conventional aerobic condition that, even with slower diffusion, the cells on PET8C manifested good viability. The serpentine morphology observed on PET8C (Figure 1C) looks similar to the “dense undulating epithelial cell sheet morphology” in Colon Chip^[9c,23]^ where a similar pore size in their PDMS porous membrane was used (7 μm in Colon/Intestine Chip vs 8 μm in this study). In those studies, the authors stated that the continuous flow was the critical factor that determined the cell morphology – when the flow stopped, the undulating morphology disappeared. Our study illustrates that similar morphology can be formed in a static culture on PET8C (Figure 1C). The depletion of glucose from the basal compartment and higher IL-8 secretion of the ALI cultures on PET8C than PET0.4 suggest that the collagen layer did not hamper glucose delivery or IL-8 secretion. The higher level of IL-8 secretion from the cells on PET8C relative to other culture conditions may indicate increased stress responses under these conditions. The higher IL-8 secretion may be induced in part by the use of a collagen scaffold. For example, previous studies demonstrated that collagen 1 had the potential to increase proinflammatory gene and protein expression.^[24]^ Further studies are needed to fully understand the physiologic state of the cells and their stress responses to the microenvironment in the current model.

We demonstrate that an apical anaerobic environment is generated in the ALI culture by our O_2_ gradient device, as previously reported for a submerged culture. Small air leaks during sample preparation may cause the initial increase of apical O_2_ level. A similar initial O_2_ increase has been observed in other studies with submerged cultures,^[9e,15]^ but it took a longer time to reduce the O_2_ below 1% in the ALI culture, probably because there would be much more O_2_ molecules to consume in the gas phase than in the solution. Nevertheless, the O_2_ level decreased and maintained an anaerobic environment after 12 h. Additionally, the cells were highly viable when cultured on PET8C, contrary to the cells grown on PET0.4 under anaerobic ALI (Figure 2) without abolishing the EdU-positive proliferating cells nor MUC2-positive goblet cells (Figure 3A), suggesting that the cells have sufficient access to O_2_ via the basal enough to maintain proliferation and cell differentiation. We note that the mucus layer in this model system was significantly thinner, and more heterogeneous than the previously reported mucus layer produced by conventional aerobic ALI (Figure 3D,G,H).^[9b]^ One of the critical differences is the medium composition. Here, the medium used during the ALI culture periods contained proliferation-promoting factors, namely Wnt-3a, R-spondin, and noggin in an L-WRN-conditioned medium to improve cell viability. These components promote proliferation and inhibit differentiation and maturation of intestinal epithelial cells.^[25]^ On the other hand, the previous study used the differentiation medium (without WRN) that promotes terminal differentiation and goblet cell maturation while inhibiting proliferation. It is also possible that the accessible O_2_ in the anaerobic ALI may be insufficient to produce thick mucus. In some intestinal inflammatory conditions where infiltrated immune cells induce hypoxia due to increased O_2_ consumption, such as ulcerative colitis, the mucus tends to be thinner than that of healthy donors.^[26]^ The resulting mucus layer obtained in this study was partially penetrable to 1 μm beads (Figure 3E,F), which is similar to the bacteria-sized bead penetrable mucus layer of germ-free mice.^[27]^ Also, interestingly, center-to-edge variation was observed with a thicker mucus layer at the edge. It is unclear what causes the variation within the sample, but it is not uncommon to observe center-to-edge variation in circular culture vessels. As in submerged cultures, the impact of surface tension forces may play a role in the greater mucus thickness near the vessel edges. Regardless, to our knowledge, only a few studies have successfully generated a thick (>100 μm) mucus layer even in the aerobic condition in vitro so far,^[9b,c]^ and this study is one of the first reports of an in vitro mucus generation in an anaerobic lumen in static culture.

The high cell viability of this new model enhanced the gut bacterial coculture capability of static culture (Figure 4A-D). Notably, total *LGG* growth (in CFU) observed in the anaerobic ALI in this study was lower than the previously reported *LGG* growth in the submerged coculture with the apical O_2_ depletion on PET0.4 (less than 10^6^ CFU in this study versus more than 10^7^ CFU in submerged culture).^[15]^ This is likely due to the reduced amount of bacteria inoculated (10^3^ CFU in this study versus ≈10^5^ CFU) and reduced apical media volume in an ALI culture (100 μL in the ALI versus 500 μL in submerge cultures). Interestingly, *LGG* grew only in the coculture, and the number of viable bacteria in the anaerobic monoculture of bacteria without human cell exposure decreased from the inoculum (Figure 4E). *LGG* is known to bind to mucus, and the mucus in vivo may support *LGG* survival. Also, as a recent study reported,^[28]^ dead host cells or host cell-derived substances may release some nutrients that LGG utilizes. We showed that our new model system supports at least two bacterial inoculation cycles (inoculation – coculture – washing per cycle) with relatively stable bacterial growth (Figure 4E). Last, an obligate anaerobe *A. hallii* was highly viable after 24 h coculture with the human colon epithelium grown on PET8C (Figure S3B, Supporting Information). We attempted to retrieve *A. hallii* for further quantification, but it was much tricker for an obligate anaerobic bacterium (*A. hallii*) than the mucus-binding facultative anaerobic bacteria *LGG*.

Our study provides a simple in vitro intestinal model system with a physiological gaseous O_2_ environment and a functional mucus layer. Other prior reports successfully recapitulated the anaerobic O_2_ environment of the intestinal lumen by flowing de-oxygenated medium across the luminal cell surface^[9a,e,29]^ or by placing the cell culture inserts into an anaerobic environment.^[9d]^ We also reported an in vitro intestinal model system with the anaerobic O_2_ environment created by utilizing cellular O_2_ consumption to deplete O_2_ in the apical reservoir.^[15,16]^ However, these models failed to provide functional mucus layers thicker than 50 μm. On the other hand, a functional mucus layer was formed in a microfluidic organ chip model^[9c]^ and a cell culture insert,^[9b]^ but these models did not possess the physiological O_2_ environment which is critical for gut bacterial coculture. Our new system supports an anaerobic O_2_ environment with a functional mucus layer simply by installing a plug without the need for fluid flow (and associated flow valves and pumps). As far as we are aware, this is the first report of an in vitro model system that implemented the two critically important features of the human colon- an anaerobic lumen and a thick protective mucus. However, more work needs to be done to fully recapitulate the host-gut microbial interactions in vitro. In our current model, longer term culture to increase the mucus thickness was not possible due to a loss of cell viability. An in vivo-like thick double-layer with a uniformly distributed bacterial impenetrable inner layer and fast replenishment is yet to be achieved in the presence of long-term epithelial-cell coculture with complex gut microbiota. Importantly, the enhancement of viability and coculture ability of the new model should be verified with the cells from multiple donors. However, we believe that this proof-of-concept study provides a promising new in vitro platform for the human intestine. Also, our study offers insights into important design factors in developing an in vitro coculture system for mimicking the human intestine and paves the way to create physiologically more relevant, more in vivo-like intestinal model systems with gut bacteria for pharmacological drug testing or screening of dietary or toxic compounds.

## 4. Conclusion

We present a new intestinal model system for gut bacterial coculture with the mucus layer in the physiological O_2_ environment. PET8C, a thin collagen layer overlaid onto a PET membrane with larger pores than the commonly used PET0.4, enhances host cell viability resulting in serpentine-shaped, undulating cell morphology. The anaerobic lumen of the in vivo colon was re-created using an anaerobic ALI culture by depleting apical O_2_ through cellular O_2_ consumption and blocking O_2_ influx to the apical side in ALI cultures. In the anaerobic ALI cultures, the human primary colonic epithelial cells properly preserved proliferative cells and goblet cells and were covered with the cell-derived mucus layer with center-to-edge variations. Our new model supports the coculture of the probiotic facultative anaerobe *LGG* and an obligate anaerobe *A. hallii*, demonstrating great potential for this gut bacterial coculture platform. Undoubtedly much more work is required to mimic the microenvironments and microbiota of the in vivo colon more closely. Nonetheless, this promising static model system partially overcame some of the drawbacks of the use of porous membranes and static culture for the epithelial cell culture models and shows the potential to offer a gut bacterial coculture platform with the microenvironment mimicking a healthy colon for pharmacological studies or screening of dietary or toxic compounds.

## 5. Experimental Section

### Preparation of PET8C:

A thin collagen layer covered polyester (PET) membrane with 8 μm sized pores was prepared by first forming collagen gel on the PET membrane and then drying it (Figure 1A). First, the porous membrane with 8 μm pores (Sterlitech #PET8025100) was treated in a plasma cleaner for 5 min (Harrick Plasma, Cat #PDC-32G). Then the plasma-treated PET membrane was treated with 5% 3-aminopropyltriethoxysilan (APTES) in water for 1 h, washed with water then 1% glutaraldehyde (GA) for 30 min, and air dried. The membrane was placed on a PDMS surface, and collagen gel was molded using a short cylindrical PDMS mold with an inner diameter of 12 mm and height of 3 – 7 mm. For the collagen gel formation, 1 mg mL^−1^ of neutralized collagen solution was prepared by mixing Rat tail collagen I solution (Corning 354 236) with neutralization buffer that contains 20 mM HEPES, 53 mM NaHCO_3_, and NaOH equivalent to the acetic acid concentration in the Rat tail collagen I solution, in PBS. 200 μL of neutralized collagen solution was added to the mold on the PET membrane and incubated for 30 min at 37 °C. Then, the PDMS mold was removed from the PET membrane leaving collagen gel on the PET membrane. The collagen gel was further dried in a 40 °C oven for at least 5 h, which resulted in a thin collagen layer covered by salt crystals. The salt crystals were removed by gently rinsing the membrane with deionized water three times.

### Preparation of the Cell Culture Inserts:

Cell culture inserts designed for the oxygen gradient^[15]^ were used to culture the contiguous colon epithelium. This insert possesses the same cell growth area and 4.615 mm shorter height as a 12-well Transwell to be used in conjunction with a 12-well plate as a basal medium reservoir.^[15]^ An insert was designed to be used in pair with a matching plug for depleting O_2_ in the apical compartment when cells are present by cellular O_2_ consumption. Porous membranes, either collagen overlaid PET membrane with 8 μm prepared above (PET8C), PET membrane 8 μm without collagen (PET8), or PET membrane with 0.4 μm (Sterlitech #1 300 016) without collagen (PET0.4), were attached to the inserts using a medical-grade double-side tape (3 M, 1504XL). Overhung residual porous membrane outside the insert wall was removed using a surgical blade. Then the inserts with the collagen-coated membrane were sprayed with 70% ethanol in a biosafety cabinet, allowed to dry, and stored dry and sterile in a 12-well plate. Immediately before the cell culture, the porous membrane was washed with sterile PBS, and then Matrigel (Corning, #354 234) was coated on top of the collagen layer by incubating 500 μL of 1% Matrigel in PBS (0.12 mg/mL at final concentration) at 37 °C for at least 30 min.

### Maintaining Primary Human Colonic Epithelial Cells:

The human colonic epithelial cells were isolated from de-identified cadaveric donors (D1- male, RRID: CVCL_ZL23 (https://web.expasy.org/cellosaurus/CVCL_ZR41), D2 – female, RRID: CVCL_ZR42 (https://web.expasy.org/cellosaurus/CVCL_ZR42) used for Figure 3A,B). The use of primary cells from de-identified cadaveric donors does not constitute human-subject research, and therefore, this study was exempted from IRB approval and consent. The cells were expanded without transformation and routinely maintained in the maintenance medium (MM, Table S1, Supporting Information) on a slab of collagen hydrogel as described previously.^[17,30]^ Briefly, the human epithelial cells were isolated from the transverse colon and cultured on collagen gel in 6-wells (1 mg mL^−1^, 1 mL per well) prepared in a CO_2_ incubator at 37 °C as described previously.^[30]^ The cells were passaged every 5–7 days by degrading collagen gel using collagenase IV (Worthington Biochemical Corporation) and dissociating the cells by 0.5 mM EDTA in PBS. The cells with passage number 6–15 were used and the absence of chromosomal abnormality was verified by karyotyping.

### Culturing Primary Human Colonic Epithelial Cells in the Cell Culture Inserts:

The primary colon epithelial cells grown in a 6-well were seeded into 6 inserts prepared above. The cells were cultured for 6 days in submerge culture in EM (composition in Table S1, Supporting Information) to form a continuous monolayer. Then from day 6, the cells were cultured in the air-liquid interface (ALI) aerobically with the basal medium (BM, refer to Table S1, Supporting Information for the composition) that contained 33 ng mL^−1^ of vasoactive intestinal peptide (VIP, AnaSpec, AS-22872) for 4 days. For post-confluent ALI cultures, a reduced amount of L-WRN (10 or 20%) titrated to make a final Wnt-3a concentration of 20 ng mL^−1^ was added to the basal medium to improve cell viability and aid cell replenishment based on the observation of the cells grown on PET0.4 in the anaerobic ALI as described earlier. VIP was added to prevent apical dehydration by regulating the movement of salt (and hence passive water movement across the membrane) to maintain a thin aqueous film across the apical cell surface.^[9]^ On day 10, the anaerobic ALI culture was initiated by installing the plug in the anaerobic chamber (Coy Laboratory) that contained 5% CO_2_ and 95% N_2_. After sealing the hole in the plug with a rubber cap, the cultures with the plug installed were brought out of the anaerobic chamber and incubated in a CO_2_ incubator at 37 °C.

### Measuring the O_2_ Level in the Apical Space in the Anaerobic ALI:

Apical O_2_ level in the anaerobic ALI was measured using an O_2_ meter (Microx4, PreSens, Germany) with a needle-type O_2_ sensor (PreSens, NFSG-PSt1) designed for gas-phase measurement. The cells were cultured for 6 days in submerge culture and then in ALI for 4 days aerobically. On day 10, a plug was installed onto a cell culture insert with post-confluent epithelial cells in the anaerobic chamber, and then the hole in the plug was sealed with a rubber cap. A needle-type O_2_ sensor was located through the rubber, sealing the hole in the plug in the middle of the apical space (≈1.5 mm from the cell surface). And the cell culture with the O_2_ sensor was brought out of the anaerobic chamber, moved to a CO_2_ incubator for incubation, and the measurement started. The O_2_ level was read every 5 min with a fixed temperature of 37 °C.

### Permeability Comparison of the Porous Membranes:

The permeability of the porous membranes was assessed by measuring the diffusion of FITC-dextran through the membrane over time. PBS and 250 μg mL^−1^ FITC-dextran (40 kDa, Sigma) in PBS were placed in the apical and basal compartments, respectively, and the fluorescence intensity was measured over the indicated time of incubation at 37 °C. FITC-dextran concentrations in the samples were estimated through a standard curve obtained from standard samples prepared at the same time. $P_{\text{app}}$ was calculated based on the equation below.

(1) $$\frac{dC_{a}}{dt}=P_{app}A\frac{C_{d}}{V_{a}}$$

here, $C_{a}$ was the concentration of the acceptor (apical) compartment, $C_{d}$ the concentration of the donor (basal) compartment, A the area of the membrane (0.85 cm^2^), and $V_{a}$ the volume of the acceptor compartment (0.5 mL). For comparison of $P_{\text{app}}$ to previous reports using reporter molecules with different molecular weight, $P_{\text{app}}$ was estimated based on the linear relation between the reciprocal of Stokes radius of the reporter molecule and $P_{\text{app}}$ derived from Einstein-Smoluchowski equation and Stokes relation.^[31]^

### Glucose Assays and IL-8 ELISA:

Glucose levels in the cell culture media were measured using Amplex Red glucose/glucose oxidase assay kit (ThermoFisher, A22189) following the manufacturer’s instruction. The basal media were collected on day 10 after 2 days (RPMI) or 1 day (Advanced DMEM/F12) of cell exposure in aerobic ALI for the glucose assay shown in Figure 1D. IL-8 levels secreted to the cell culture media were measured using an IL-8 ELISA kit (ThermoFisher, #88-8086-22) following the manufacturer’s protocol. Basal media with 2 days of cell exposure in aerobic ALI were collected on day 10 and used for IL-8 ELISA.

### Cell Viability Assessment:

To estimate the cell viability, 1 μM Calcein-AM, propidium iodide (PI), and Hoechst 33342 were added to the basal medium and incubated for 30 min at 37 °C to label the cytoplasm of live cells, DNAs of the membrane-compromised dead cells, and all DNAs respectively. In the anaerobic ALI condition, the plug remained in place during staining. Then the cell culture inserts were taken out of the well plate, place on glass slide and labeled live cells were imaged using a laser scanning confocal fluorescence microscope (Olympus, Fluoview 3000) with 561 nm, 488 nm, and 405 nm lasers for exciting PI, Calcein-AM, and Hoechst 33342, respectively. The emission was collected at 610–710, 500–540, and 430–470 nm, respectively with PMT detectors.

### Assessment of Cell Phenotypes:

S-phase cells were labeled by incubating thymidine analog 5-ethylyl-2′-deoxyuridine (EdU, Lumiprobe, Cat #10430, 10 μM) for 24 h with the cells. The cells were fixed with 4% paraformaldehyde in PBS for 15 min and permeabilized with 0.5% Triton-X 100 in PBS for 20 min at 25 °C. Then, the fixed cells were washed with 3% bovine serum albumin (BSA), and EdU incorporated into the cellular DNA was detected by conducting a click reaction with sulfo-cyanine azide (2 μg mL^−1^, Lumiprobe Cat #A3330), CuSO4 (2 μM) and sodium ascorbate (40 mg mL^−1^) in PBS for 1 h at 25 °C. The cells were then washed once and blocked with 3% BSA in PBS for 1 h at 25C. Mouse primary antibody for MUC2 (Santa Cruz Biotechnology, sc-7314, RRID: AB_627 970) was diluted at 1:250 in 3% BSA in PBS and incubated with the cells for 16 h at 4 °C. Then, the primary antibody was removed, and the cells were washed with 3% BSA three times and incubated with anti-mouse secondary antibody conjugated with Alexa Fluoro 488 (Jackson ImmunoResearch Labs, Cat # 115–545003, RRID: AB_2338840) diluted at 1:500 in 3% BSA in PBS that contained Hoechst 33 342 (Thermo Fisher, Cat # H1399, 2 μg mL^−1^) for 1 h at 25 °C. Finally, the labeled cells were washed once with 3% BSA and then with PBS.

The stained cells were imaged using a laser scanning confocal fluorescence microscopy (Olympus, Fluoview 3000) with an excitation of 640 nm, 488 nm, 405 nm, and emission of 650 – 750 nm, 500 – 540 nm, and 430–470 nm for EdU, MUC2, and Hoechst 33 342, respectively. The entire stained area of samples was imaged, stitched, and subjected to quantification. The images were subjected to manual thresholding to identify the stained area for each marker using Fiji software,^[32]^ and the positive area for EdU and MUC2 each was divided by Hoechst 33342 positive area for Figure 3.

### Mucus Thickness Measurement:

The mucus thickness was assessed using 1 μm-sized fluorescent beads (ThermoFisher, F13083). 2–5 ×10^6^ beads in HBSS were gently added to the apical side. Cell cytoplasm was labeled using Calcein AM. Then the cell culture insert with the plug was placed on a glass slide and Z-stack images were taken using a confocal fluorescent microscope (Olympus, Fluoview3000). The fluorescent beads and the Calcein-AM stained cells were excited using a 561 nm and a 488 nm laser, respectively, and the emissions were collected with PMT detectors (610–710 nm for the beads and 500–540 nm for the cells). Locations of the beads were extracted from the z-stack images using CellProfiler 4.2.0.^[33]^ Mucus thickness was calculated by subtracting the cell height (estimated from Calcein-AM intensity data) from the locations of the beads. The cell height plane was defined for each pixel by identifying the z location where the fluorescence intensity becomes 20% of the maximum intensity and then using a Gaussian filter with a sigma value of 15 to minimize overestimating cell height on noise signals.

### Bacterial Culture and Inoculation for the Coculture:

*Lactobacillus rhamnosus GG* (ATCC53103, purchased from Microbiologics, #01090P) was cultured aerobically in De Man, Rogosa, Sharpe (MRS) medium (BD, #288 130) at 37 °C. For coculture with the human colon epithelial cells under the anaerobic ALI, a single colony of *LGG* was cultured in an MRS medium for 16 h prior to the coculture. For coculture, 1000 CFU of *LGG* (0.1 mL of 10^4^ CFU mL^−1^) in 10% peptone yeast glucose (PYG) medium (peptone 10 g L^−1^, yeast extract 10 g L^−1^, dextrose 5 g L^−1^, resazurin 1 mg L^−1^, L-cysteine·HCl 0.5 g L^−1^, CaCl_2_·H_2_O 100 mg L^−1^, MgSO_4_·7H_2_O 50 mg L^−1^, K_2_PO_4_ 40 mg L^−1^, KH_2_PO_4_ 40 mg L^−1^, NaHCO_3_ 0.4 g L^−1^, NaCl 80 mg L^−1^, hemin (5 mg L^−1^) and vitamin K (1 mg L^−1^)) diluted in Hank’s Balanced Saline Solution was inoculated in the anaerobic chamber on the apical side through the hole of the plug installed for apical O_2_ depletion. To enumerate CFU/mL at t = 24 h of coculture, the supernatants and the first wash of the coculture were collected and plated aerobically on PYG agar plates with serial dilution, and the colonies were counted after 1–2 days.

### Statistical Analysis:

Statistical analysis was performed using Graph-Pad Prism 9. The detail of each analysis including the types of statistical analyses and the number of technical replicates (n) was specified in the figure captions. Mostly, ordinary unpaired Brown-Forsythe and Welch ANOVA tests (which do not assume the same standard deviations of the two groups to compare) were used to compare the means of the data from PET 4 and PET8C samples and calculate p values. For Figure 3B, where two biological replicates were used, unpaired Welch’s t-test was performed on all data since the data from different donors were not significantly different.

### Ethics Approval Statement:

The human primary cells used in this study were obtained from de-identified cadaveric intestines. The use of primary cells from de-identified cadaveric donors does not constitute human-subject research, and therefore, this study was exempted from IRB approval, which was consistent with the position statement and definition of human-subject research and use of cadaveric tissue by the National Institutes of Health (https://humansubjects.nih.gov/from-applicants).

## Supplementary Material

SUPINFO

## Figures and Tables

**Table 1.** Porous membranes used in this study.

	PET0.4^a)^	PET8^b)^
Pore size	4 μm	8 μm
Pore density	2 × 10^6^ cm^−2^	1 × 10^5^ cm^−2^
Open area	0.3%	5%

a) From the manufacturer’s information for Sterlitech #1300016;

b) From the manufacturer’s information for Sterlitech #PET8025100.

## Data Availability

The data that support the findings of this study are available from the corresponding author upon reasonable request.
