# Supplementary material for: A Microphysiological System with an Anaerobic Air-Liquid Interface and Functional Mucus Layer for Coculture of Intestinal Bacteria and Primary Human Colonic Epithelium
Source: Adv Mater Interfaces. Author manuscript; Available in PMC 2025 Sep 3. (PMC11460523; doi:10.1002/admi.202400093)
Supplement: SUPINFO [file NIHMS2002284-supplement-SUPINFO.docx]

**Supplementary Information**

**A Microphysiological System with an Anaerobic Air-Liquid Interface and Functional Mucus Layer for Coculture of Intestinal Bacteria and Primary Human Colonic Epithelium**

*Raehyun Kim*^1^**, Nancy L. Allbritton*^2^***

Raehyun Kim

Department of Biological and Chemical Engineering, Hongik University, Sejong, Republic of Korea

E-mail: raehyunkim@hongik.ac.kr

ORCID ID: 0000-0002-8796-2004

Nancy L. Allbritton

Department of Bioengineering, University of Washington, Seattle, WA, USA

E-mail: nlallbr@uw.edu

ORCID ID: 0000-0002-9242-768X

Table S1. Medium formulation

|  | Maintenance medium (MM) | Expansion medium (EM) | Basal medium (BM) |
| --- | --- | --- | --- |
| Advanced DMEM/F12  (Thermo Fisher) | 50% (v/v) | 50% | 90% or 80%*^2^ |
| L-WRN conditioned medium | 50% (v/v) | 50% | 10% or 20%*^2^ |
| GlutaMax  (Thermo Fisher) | 1x | 1x | 1x |
| HEPES | 10 mM | 10 mM | 10 mM |
| Human EGF or mouse EGF (for Medium 2 only) (Peptrotech) | 50 ng/mL | 50 ng/mL | 50 ng/mL |
| B27 | 1x | 1x |  |
| N-acetyl cysteine  (MP bio) | 1.25 mM | 1.25 mM |  |
| Gastrin  (Anaspec) | 10 nM | 10 nM |  |
| Y-27632  (ApexBio) | 10 μM | 10 μM*^1^ |  |
| A83-01  (Sigma Aldrich) | 500 nM |  | 500 nM |
| Prostaglandin E2 (PGE2)  (Cayman chemicals) | - | 10 nM |  |
| Nicotinamide  (Sigma Aldrich) | - | 10 mM |  |
| SB202190  (Selleckchem) | 3 μM | 3 μM |  |
| Primocin  (InvivoGen) | 50 μg/mL | 50 μg/mL |  |
| FBS  (HyClone, heat inactivated) | - |  | 10% |

*1. It was added for the first 2 days of the culture.

*2. The amount of L-WRN was titrated to make 20 ng/mL at the final concentration and the volume of the Advanced DMEM/F12 was adjusted accordingly. The concentration of the Wnt-3a was measured by ELISA (Mouse Wnt-3a DuoSet ELISA, R&D Systems)


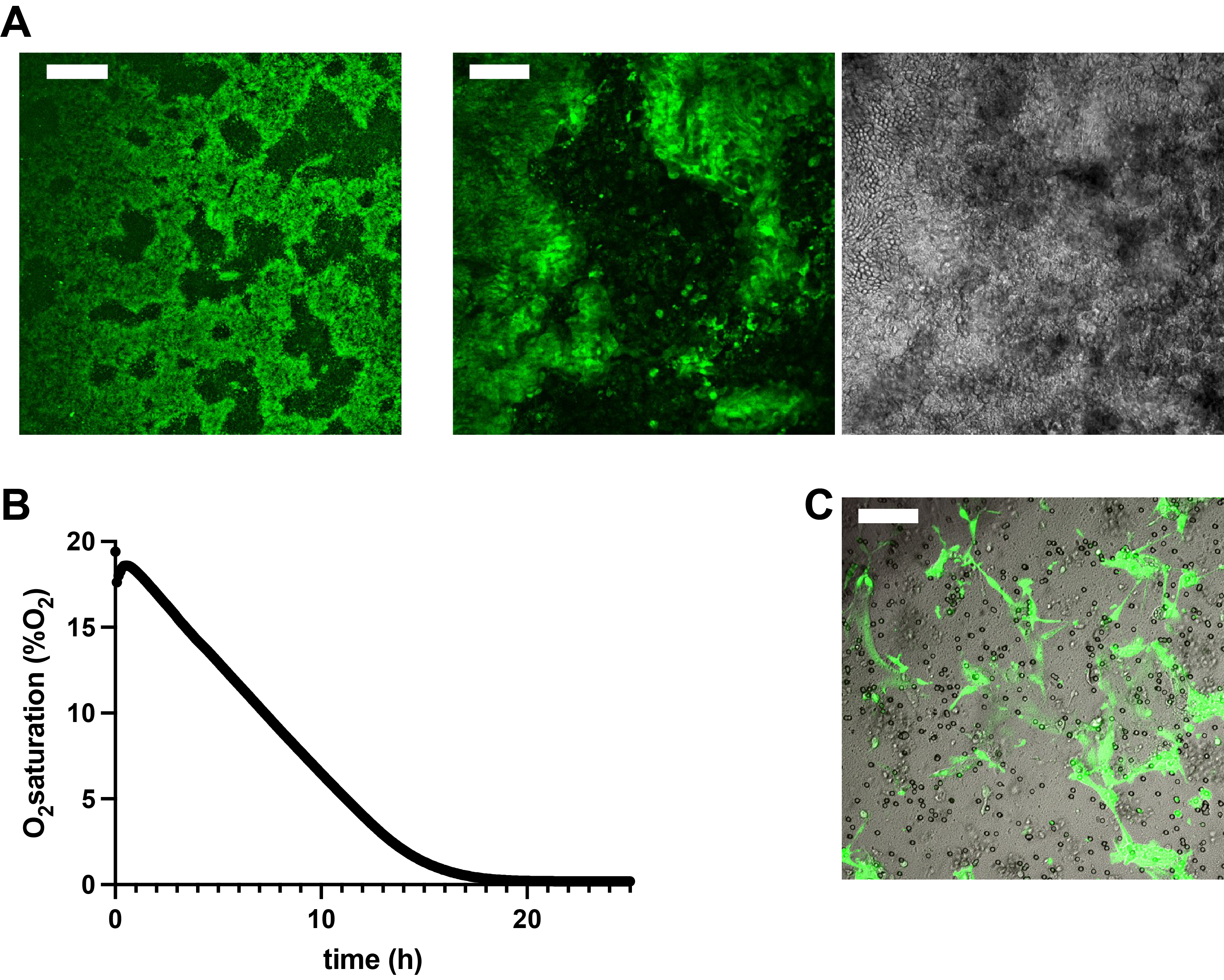


**Figure S1.** A) The cells cultured on PET0.4 were exposed to 2 days of an anaerobic ALI in the differentiation medium. Live cells were labeled with Calcein-AM (green). The left and right images show the same sample in a low resolution (left) and a higher resolution (right). Scale bar: left 500 μm, right 100 μm. B) A representative data of the apical O_2_ profile from the cells cultured on PET0.4 in the O_2_-depleting cell culture cassettes. The cells were cultured post-confluent in aerobic ALI for 4 days (without plug) then subjected to apical O_2_ depletion by installing the plug. C) The cells were cultured on PET8 without a collagen layer. Live cells were labeled with Calcein-AM. Scale bar: 100 μm.


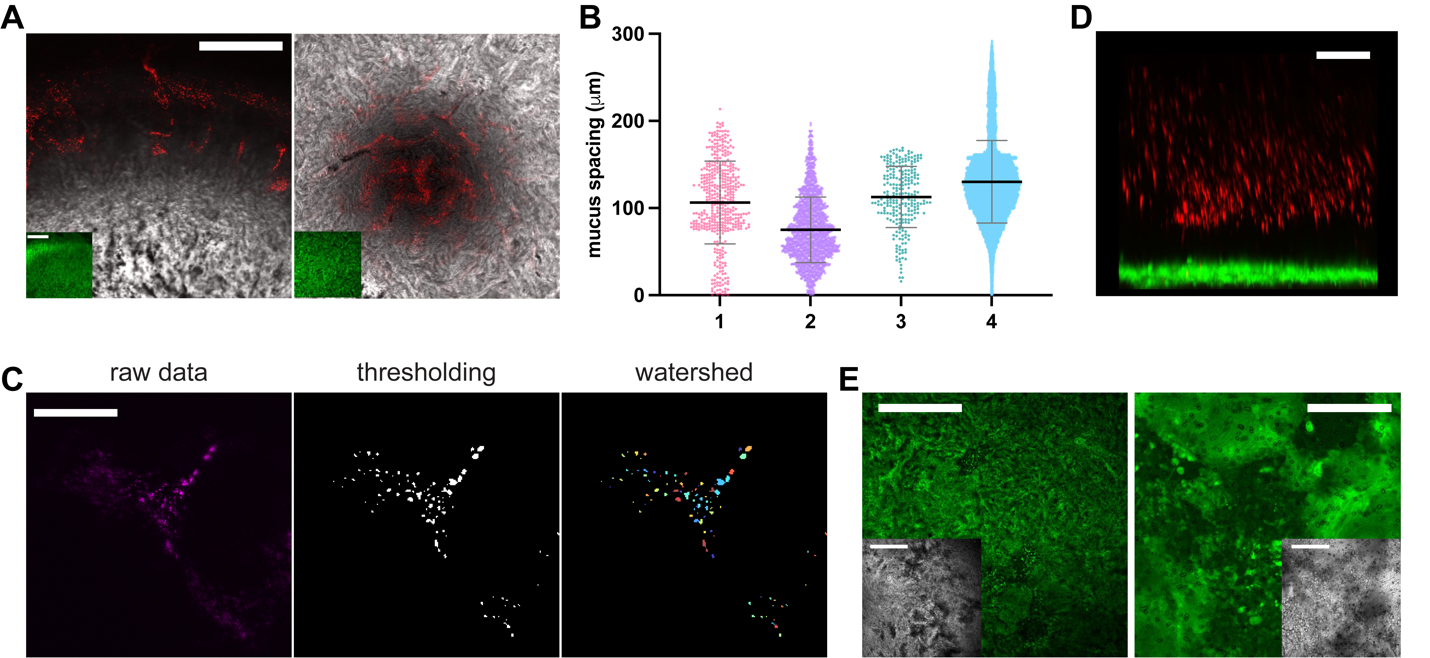


**Figure S2.** A) Representative images of the fluorescent beads on the human colon epithelial cells (top view). Scale bar: l mm. B) The relative locations of the fluorescent beads from 4 independent cultures. Each spot corresponds to a bead at the location detected relative to the cell surface. At least 240 beads from 2-4 areas per sample were imaged. 1 is the same data as Figure 3F for a comparison. C) Representative images of processed fluorescence signals of the fluorescent beads for Figure 3C “Adv. DMEM/F12 edge” using CellProfiler. The scale bar = 200 μm. D) Representative xz projection images of beads overlaid on the cells cultured on PET8C without L-WRN conditioned medium in the apical anaerobic ALI. E) Representative images (top view) of the cells at two different locations in the same sample exposed to 2 days of anaerobic ALI in RPMI base medium supplemented with 20% L-WRN. Green indicates Calcein-AM labeled live cells, and grey inset transmitted DIC images. Scale bar: 1 mm, for insets 200 μm.

**
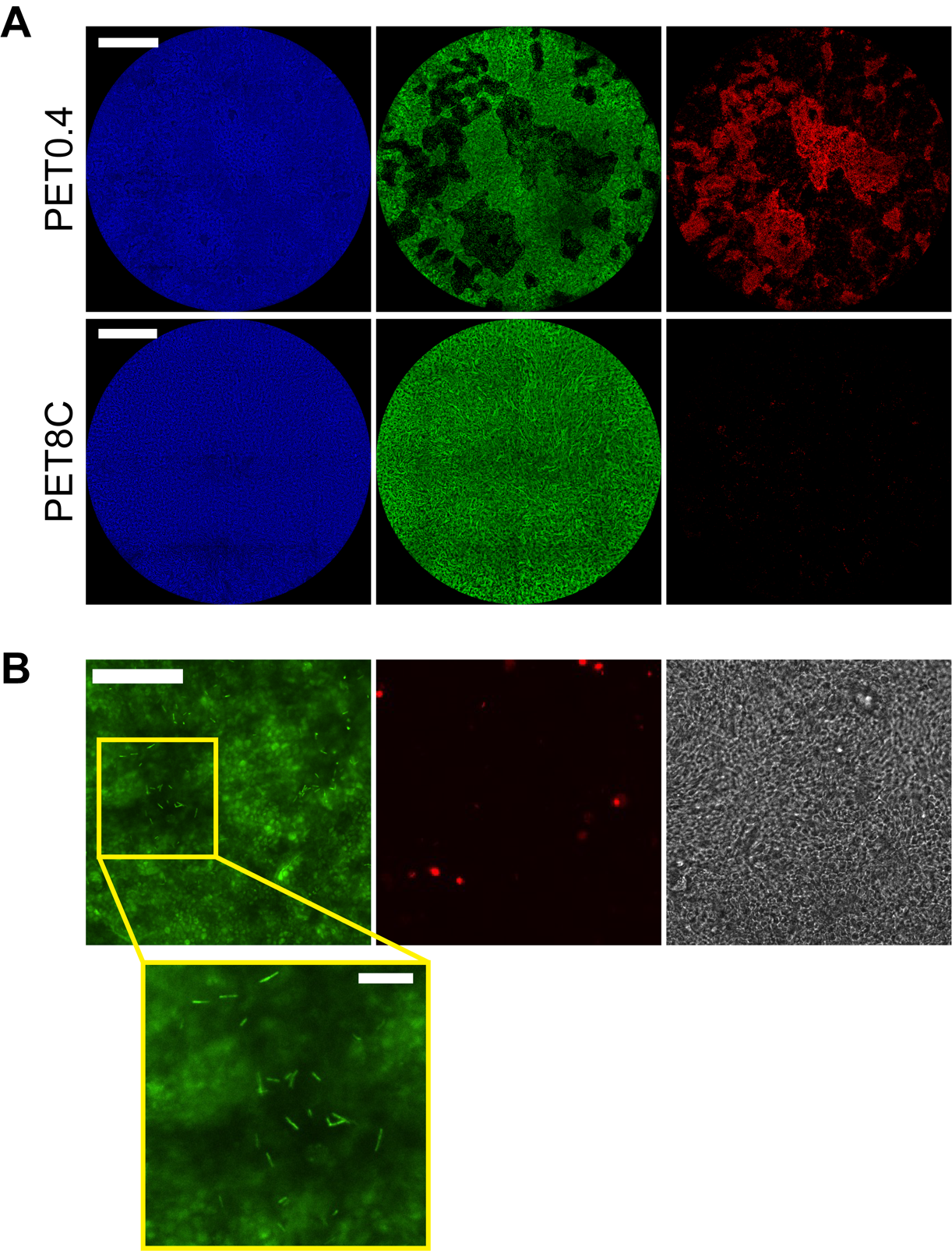
**

**Figure S3**. A) Representative images of the human colon epithelium cultured on PET0.4 and PET8C after 1 day of *LGG* coculture. 3182 μm by 3182 μm images were stitched together. Blue, green, and red indicate Hoechst 33342, Calcein, and PI, respectively. Scale bar = 2 mm. B) Representative images of the *A. hallii* coculture with the human colon epithelium cultured on PET8C under the O_2_ gradient. Green: nuclear stain Syto9, red: PI, grey: DIC. The scale bar indicates 100 μm for the larger image and 25 μm for the blowup image.
